# Nemato-toxic analysis of several chopped plant leaves against Meloidogyne incognita affecting tomato In vitro and In pots

**DOI:** 10.6026/97320630018354

**Published:** 2022-04-30

**Authors:** Mohd Ikram, Mohammad Shariq, Faryad khan, Arshad Khan, Saba Fatima, Mansoor A Siddiqui

**Affiliations:** 1 Section of Plant Pathology and Nematology, Department of Botany, Aligarh Muslim University, Aligarh 202002, India

**Keywords:** Botanicals, Meloidogyne incognita, nematicidal efficacy, root-knot disease, LC-50

## Abstract

Tomato plant is affected by several pathogens, including root-knot nematodes (RKNs), belonging to the genus Meloidogyne. Meloidogyne incognita is among the most potent pests infecting tomato roots. Therefore, it is of interest to discuss the management of Meloidogyne incognita using selected botanicals such as Cammelina benghalensis, Evolvulus nummularius, Gomphrena celosioides, Lindenbergia indica, Scoparia dulcis and Vernonia cinerea. The second-stage juveniles (J2s) of M. incognita were directly treated with the aqueous extracts of the botanicals at varied concentration ranging from 10-100%. 100% concentration of Lindenbergia indica was found to be the most toxic against the survival of J2s of M. incognita as compared to other concentrations. In vitro tests also showed the maximum inhibition in egg hatching at 100% concentration after seven days in the extract of Lindenbergia indica. Moreover, botanicals significantly reduced the infestations in relation to number of root galls, eggmasses/root and nematode population/250 g soil in pots. The plant treated with Scoparia dulcis leaves showed the highest nematicidal efficacy with maximum reductions in all the pathological parameters as compared to the untreated control. All treatments resulted in increased growth, physiological parameters and decreased pathological parameters of tomato.

## Background:

Tomato (Solanum lycopersicum L.) is a member of the family Solanaceae and cultivated commercially around the world. It is a good source of vitamins, minerals and contains essential carotenoids including lutein, lycopene natural antioxidants that help to regulate blood pressure, protect against cancer, and decrease blood glucose levels in diabetic people [1]. India is the second most prominent producer of tomato in world. However, the production of tomato in terms of quantity and quality is severely affected with the pathological effect of pests. Root knot nematodes (Meloidogyne spp.) are one among the major pathogen affecting tomato growth and severely effect its physiology. Meloidogyne incognita affects the crop yield directly and makes the plants more susceptible to various bacterial and fungal infections [2]. Plant-parasitic nematodes attack a wide range of cultivated crops, causing estimated annual yield losses of more than EUR 157 billion [3]. The estimated yield loss of tomatoes caused by nematodes is about 23% in India [4]. Meloidogyne spp. is one of the most economically damaging horticultural pests, causing an estimated yearly loss of US$ 100 billion globally [5]. They are difficult to control due to their short life span, large population density and high reproductive capacity [6]. Various methods have been employed to manage nematodes disease, including chemical, physical, organic amendments, biological control and cultural practices [7,8]. Applying chemical nematicides is a common, effective and widespread tactic to manage nematode infestations and minimize productivity losses [9]. Nevertheless, these chemicals enhance biodegradation and environmental pollution for a long time and persist negative impact on flora and fauna [10]. Alternative nematode management strategies are highly needed because of these harmful nematicides' limits. Botanical amendments may be used as an alternative tactic over chemical nematicides. It may serve a dual purpose in pest control and soil nutrition enhancement. Botanicals release a lot of biologically active secondary metabolites (viz., phenolics, alkaloids, glucosinolates, flavonoids, isothiocyanates, terpenes, sesquiterpenes, polyacetylenes and thienyls) after their degradation in the soil that have been extensively explored for antagonistic nematode properties [11]. In the present time, secondary metabolite and plant by-products have gained great attention for managing plant-parasitic nematodes and other phytopathogens [12]. Numerous plants such as Parthenium hysterophorus, Cymbopogon citratus, Eichhornia crassipes, Monstera deliciosa and Tinospora cardifolia were already used to manage plant-parasitic nematodes [13]. Therefore, it is of interest to evaluate the effect of various plant leaves against M. incognita infection in tomato in vitro and pots conditions.

## Materials & methods:

### Collection of inoculum and maintenance:

Infected roots were collected from the eggplant field from village Panjipur, district Aligarh. Eggmasses were detached from the infected root and collected in a petri dish distilled water. Meloidogyne spp. was identified by perineal patterns in the laboratory [14]. After identification, a single eggmass was cultured and maintained on eggplant in the greenhouse of the Department of Botany, Aligarh Muslim University, Aligarh. Eggmasses were hand-picked using sterilized forceps from heavily infected roots of eggplant. The second stage juveniles (J2s) were obtained from hatched eggs by incubating hand-picked egg masses in sterile distilled water at 27 ± 2°C. The hatched juveniles were collected every 24 hours, and distilled water was added. The concentration of freshly hatched second-stage juveniles was standardized.

### In vitro experiment:

#### Preparation of botanical extract:

The leaves of six botanical species screened their nematicidal properties. Cammelina benghalensis, Evolvulus nummularius, Gomphrena celosioides, Lindenbergia indica, Scoparia dulcis, and Vernonia cinerea were collected from Aligarh Muslim University, campus. These plants were immediately brought to the laboratory, and each plant was washed with tap water and blotted with filter paper. The selected leaves were cleaned and dried carefully at 58°C for 48 hours in an oven. And to use a clean grinder, the dry materials were ground into powder. 10 gm powder of each plant product was taken and dissolved in 1 liter of sterile distilled water and left for 24 hours. All extracts were filtered using Whatman No.1 filter sheets to eliminate debris. A stock solution of 100 % was prepared in distilled water. Different concentrations as; 75%, 50%, 25%, and 10% were made by dissolving the required amount of distilled water.

#### Eggs hatching test:

To the hatching experiment, five healthy uniform size eggmasses of M. incognita were picked up from the infected brinjal plant root and placed in Petri dishes, each containing 10 ml of different concentrations of six plant extract. Petri dishes containing five egg masses in distilled water were employed as a control. For seven days, all of the Petri plates in the laboratory were left at room temperature to allow the eggs to hatch. Each treatment was carried out five times. A binocular microscope was used to count the number of hatching juveniles with the help of a counting dish.

#### Juveniles’ mortality test:

For the in-vitro mortality test, 120 freshly hatched second-stage juveniles of M. incognita present in 0.2 ml water are transferred to 9.8 ml different concentrations of each Plant extract in Petri plates. Double Distilled Water in Petri dishes served as control. Each treatment was replicated five times. The number of living and dead juveniles was counted using a binocular microscope following incubation periods 24, 48, and 72 hours. The nematodes that showed any motion were regarded as alive, but the worms that did not show any mobility and had a straight body shape were considered dead. The total number of dead and alive nematodes was counted. The data on concentrations and mortality rates were analysed and the LC-50 values for all treatments were calculated.

The formula was used to quantify the percent inhibition in egg hatching or mortality.

% inhibition or mortality= Co - Tα/Co x 100%

Where, in case of egg hatching, C0 = number of juveniles hatched in control

Tα = number of juveniles hatched in each concentration of extract, In case of mortality C0 = number of live nematodes in the control petri dish

Tα = number of live nematodes after 24-, 48- and 72-hours exposure.

### Pots experiment:

The pot study was carried out in a greenhouse at Department of Botany, Aligarh Muslim University, Aligarh. Six-inch clay pots were filled with 1 kg autoclaved soil in a 3: 1 ratio (sandy loam: farmyard manure). The soil was amended with 30 gm of freshly cut leaves from the tested plant. The soil of each pot, also amended with 5 gm dry powder of leaves of the Lindenbergia indica. The required amount of water was given regularly into the pots for proper decomposition of freshly chopped leaves. The seeds of the tomato cultivar Pusa-Ruby were purchased from IARI, New Delhi. The seeds were surface-sterilized in 0.01 percent HgCl2 for two minutes before being washed three times with Double Distilled Water (DDW). Surface sterilized seeds sown in pots for prepared nursery. Single healthy seedlings were transferred from nursery to each treated pot, including control and properly maintained the pots. Each pot was inoculated with 1500 freshly hatched second-stage juveniles of M. incognita by making holes in the rhizosphere. A fully randomized design (CRD) was used in the trial, with five replications of each treatment and control. Untreated uninoculated and inoculated plants were taken as control. Throughout the experiment, pots were supplied with the needed amount of water on a regular basis.

### Observation and data collection:

Ninety days after inoculation, tomato plants were uprooted from their pots and roots were cut from the shoot. To avoid eggmasses being damaged, the roots of each tested plant were carefully cleaned in a bucket of water. The data were collected as growth, yields, physiological and pathological parameters such as shoot and root lengths, fresh weights, dry weights, chlorophyll, carotenoid contents, nitrate reductase activity (NRA), Proline, number of galls, eggmasses/root, and nematode population/250 gm soil. The population of the root-knot nematodes was assessed by Cobb's sieving and decanting technique [15 - check with author].

### Estimation of chlorophyll and carotenoids content:

The chlorophyll and carotenoid content of the fresh leaves was determined by Mackinney's method. One gm of fresh leaves detached from the plant and thoroughly ground with a mortar and pestle. After that, add 20 mL of 80% acetone to the pulp. After centrifuging the mixture at 5000 rpm for 5 minutes, the supernatant was collected in a volumetric flask. The Residues were washed three times with 80 percent acetone, each time using the same volumetric tube and the final volume was labelled with 80 percent acetone. A spectrophotometer (Shimadzu UV-1700, Tokyo, Japan) was employed to analyse absorbance at 645 and 663 nm for chlorophyll and 480 and 510 nm for carotenoid beside the blank (80% acetone). The chlorophyll and carotenoid content of the extract (mg g−1 tissue) was estimated using the equation below.

Total chlorophyll content = 20.2(A_645_)+8.02(A_663_) x V/ W x D x 1000

Carotenoid content = 7.6(A_480_) + 1.49(A_510_) x V/W x D x 1000

Where, A480, A510, A645, A663 = Absorbance of extract at given wavelengths (480, 510, 645, and 663 nm, respectively), V=Final volume of the extract, W=Fresh weight of leaf sample D=Length the path of light.

### Estimation of Proline content:

Proline content in leaf tissues was determined using a ninhydrin reaction [16]. For this purpose, 0.25gm leaf sample was grinded in 5 ml of 3% sulfosalicylic acid with the help of a mortar pistil. This sample was centrifuged at 10000 rpm for 10 minutes. 1ml supernatant, ninhydrin acid and glacial acetic acid (1:1:1) was incubated at 90°C for 1 hour to colorimetric measurements. The reaction accrued then cooled in an ice bath. After this, 2ml of toluene was added and vigorously shake the sample. A chromophore was extracted, and its absorbance was measured at 520 nm using a spectrophotometer (UV 1700, Shimadzu, Japan).

### Estimation of Nitrate reductase activity (NRA):

[17] Jaworski (1971) technique was used to calculate nitrate reductase activity in fresh leaves. Each sample received 200 mg of chopped leaves, which were put to plastic vials. 2.5 ml of phosphate buffer pH 7.5 and 0.5 ml of potassium nitrate solution were added to each vial, followed by 2.5 ml of 5 percent isopropanol. These vials were incubated for 2 hours at 28 ±2°C in the dark in a BOD incubator. For the colour development, 0.3 ml of sulphanilamide solution and NED HCl were added to 0.4 ml of the incubated mixture in a test tube and left for 20 minutes. 5 ml. Distilled water was used to dilute the mixture. A spectrophotometer was used to measure absorbance at 540 nm (UV 1700, Shimadzu, Japan). A blank was run simultaneously with each sample. Using known graded concentrations of NaNO2 (sodium nitrite) solution, a standard curve was plotted. The absorbance of each sample was compared with that of the calibration curve and NRA activity (µ mole NO2 (FW) g−1 h−1) was calculated.

### Statistical analysis:

Data of hatching and mortality presented are mean values. Experimental data of pot experiment was analysed by one-way analysis of variance (ANOVA) using SPSS-17.0 statistical software (SPSS Inc., Chicago, IL, USA). The differences between treatments were determined by Duncan's Multiple Range Test. Means values were considered significant at P < 0.05.

## Results:

### Effect of extract on juveniles' mortality:

The results shown in Table 1(see PDF) reveal that mortality of second-stage juveniles of M. incognita was found nill in distilled water (control). However, different concentrations of aqueous extracts of leaves, i.e., Cammelenia benghalensis, Evolvulus nummularius, Gomphrena celosioides, Lindenbergia indica, Scoparia dulcis, and Vernonia cinerea show significant effects on juveniles (J2) mortality. The mortality of juveniles in various concentrations was shown to be directly proportional to concentration and period of exposure. The result indicates in table1 that the maximum mortality (82%) was found in the extracts of Lindenbergia indica at 100 % concentration after 72 hours of the exposure period and minimum mortality (11%) was found in Evolvulus nummularius at 10 % for 24 hours exposure period. The data of the in-vitro nematicidal performance of botanicals have given as the LC50 value with the corresponding 95 percent confidence limit. A probit result of the effect of plant extracts, including LC50, was calculated. The higher concentrations of botanical increases mortality of juveniles as 100 % concentration were shown highest mortality. The extract of L. indica was found highly toxic to juvenile mortality with LC50 values 49.893, 34.198 and 22.484 after 24, 48, and 72 h of exposure time, respectively. The results indicate that L. indica was most lethal to juveniles after 72 hours periods of bioassay. It was followed by Scoparia dulcis, Vernonia cinerea, Cammelina benghalensis, Gomphrena celosioides while aqueous extract of Evolvulus nummularius was found least toxic against juveniles of M. incognita with LC50 values, 185.884, 128.072 and 79.405 after 24, 48 and 72 hours of bioassays period, respectively (Table 2 - see PDF).

### Effect of extract on hatching of juveniles from eggs:

The aqueous leaf extracts of all the botanicals examined were considerably efficient against M. incognita egg hatching. Botanical, Lindenbergia indica was found highly effective and Evolvulus, nummularius found least effective after seven days exposure period. There were minimum eggs hatching accrued in the extract of lindenbergia indica (90.02, 159.65, 226.43, 301.01, 407.34) and maximum hatching found in the extract of Evolvulus nummularius (287.61, 340.00, 373.05, 440.07, 522.04) at the different concentrations viz., 100%, 75%, 50%, 25% and 10% compared to control (620). Results represent that maximum percent inhibition in egg hatching showed by Lindenbergia indica (85.48%) at 100% concentration (Table 3 - see PDF).

### Effect of fresh chopped leaves in plant growth parameter:

The amendment of fresh leaves of the selected plant in the soil of the pot effectively eliminates the reproduction and development of M. incognita and suppresses the number of galls, eggmasses/root and nematodes population. Thus, the botanical amendment increases the plant's yield and reduces the nematode infection from the plant. The plant growth is significantly induced by the use of chopped leaves as soil amendment. The highest shoot length (38.2cm), root length (12.4cm), fresh shoot weight (36.7g), dry weight (10.35 g), fresh root weight (4.96g) and dry weight (2.62g) were recorded in plants treated with amendment the leaves of Scoparia dulcis compared to the untreated inoculated controls (Table 4 - see PDF). The tomato plant treated with Scoparia dulcis was found maximum increased plant length as shoot (38.2) over untreated uninoculated control was recorded followed by Vernonia cinerea (36.8cm), Cammelina benghalensis (35.2cm) and Gomphrena celosioides (34.4cm) while Evolvulus nummularius (31.6cm) amendment of leaves shows minimum increment of plant length (Table 4 - see PDF). The effect of the soil amendment of all botanical was also observed in the fresh and dry weight of the plant. All treatments found prominent in enhancing the fresh weight of plants as compared with control. The maximum fresh weight was found treated plant with Scoparia dulcis (36.75g) followed by Vernonia cinerea (35.10g), Cammelina benghalensis (32.80g), Gomphrena celosioides (31.53g) and Evolvulus nummularius (30.12g) to compare against untreated inoculated control as (26.30g). Consequently, similar trends were observed in fresh weight of root as treatments of Scoparia dulscis indicate maximum fresh weight of root (12.35 g) followed by Vernonia cinerea (11.84g), Cammelina benghalensis (11.30g) and Gomphrena celosioides (10.45g), whereas Evolvulus nummularius (9.74g) found minimum enhanced the fresh weight of root. The plant treated with Scoparia dulcis, were found maximum dry weight of shoot (10.35g) and root (2.62g) while Evolvulus nummularius indicated minimum dry weight of shoot (8.56g) and root (2.18g).

### Effect of fresh chopped leaves on physiological parameters:

Infection with nematodes reduced chlorophyll content, carotenoid content, and nitrate reductase activity in the same way that it reduced plant growth variables. The use of chopped leaves increased the contents of these parameters. The result revealed that application of leaves of Scoparia dulcis increase maximum chlorophyll content (1.87 mg/g), carotenoid content (0.583 mg/g) and nitrate reductase (0.243 µmol g−1 h−1) followed Vernonia cinerea, Cammelina benghalensis and Gomphrena celosioides by whereas the minimum increase in these parameters (1.52, 0.515 and 0.198 µmol NO2 (FW) g−1 h−1) were detected in plants treated with freshly chopped l eaves of Evolvulus nummularius in the comparison of untreated inoculated control (Table 5 - see PDF). Table 5(see PDF) indicated that tomato plant treated with chopped leaves of Scoparia dulcis amendment show minimum increases the proline content (0.290) whereas Evolvulus nummularius treated plant show the proline content (0.432) compared to an inoculated control.

### Effect on yield:

It was found that yields also significantly influenced by the treatment of botanicals. Treatment with Scoparia dulcis chopped leaves observed maximum yield/plant (256g), whereas Evolvulus nummularius show minimum increment in the yield/plant (205g). Botanicals, Vernonia cinerea, Cammelina benghalensis and Gomphrena celosioides show yield/plant follow as 243g, 232g, and 214g, respectively (Table 4 - see PDF).

### Effect on number of galls, eggmasses, Eggs /eggmass and nematode population:

Table 5(see PDF) revealed that soil amendment of the chopped leaves of Scoparia dulcis, was most effective to the suppress effect of nematodes among the all-selected botanicals. The amendment of Scoparia dulcis indicate least number of galls in the roots was recorded i.e. (78) followed by Vernonia cinerea (85), Cammelina benghalensis (89), Gomphrena celosioides (95), while Evolvulus nummularius @ 30gm/pot was found minimum effective against nematode with maximum number of galls (98) as compared to untreated inoculated control (136). The minimum number of eggmasses (92) was observed in Scoparia dulcis, followed by Vernonia cinerea (97), Cammelina benghalensis (106), Gomphrena celosioides (115) while Evolvulus nummularius (124) was found maximum number of eggmasses on the roots of plant as compared to control (160). In the same context, the use of Scoparia dulcis leaves was shown to be the most significant. (P≤0.05) reduction in nematode population (1012), followed by Vernonia cinerea (1107), Cammelina benghalensis (1165), and Gomphrena celosioides (1218) while least reduction was observed in Evolvulus nummularius (1329) compared to untreated inoculated control (1680) Table 5(see PDF). Results from Table 5(see PDF) shows that a significant (P ≤ 0.05) reduction in eggmasses, eggs/eggmass nematode population and number of galls was found in all the treated pots. The Table 5(see PDF) indicated that Scoparia dulcis show a maximum reduction (@ 92 @139 @1012 @78) whereas Evolvulus nummularius show minimum reduction (@124 @186 @1329 @102) in these parameters.

## Discussion:

According to the findings, the nematicidal ability of selected plant extracts is due to certain phytochemicals. Existing research shows that many plants or their derived secondary metabolites or phytochemicals have nematicidal potential against a wide variety of plant-parasitic nematodes, including root-knot nematodes, M. incognita [20,21,22]. This in-vitro study found that the aqueous extracts of Lindenbergia indica, Scoparia dulcis, Veronia cinerea, Cammelina benghalensis Gomphrena celosioides and Evolvulus nummularius showed significant nematicidal efficacy against juvenile's mortality and egg hatching of M. incognita. Among all botanical extracts evaluated, the leaf extracts of Lindenbergia indica and Scoparia dulcis were shown to be the most efficient in lowering egg hatching and juvenile mortality. The result of in vitro suggested that Lindenbergia indica was most effective against nematode. So, to enhance the suppression effect of other botanical against nematodes, the powder of the Lindenbergia indica (5 gm) was amended in the soil of each pot, in starting the experiment excluding control. The nemato-toxic potential was found to be directly related to the extract concentration, i.e., the higher the concentration, the larger the nemato-toxic potential, and vice versa. These results are in agreement with [23, 24] Saeed et al. 2015; Khan et al. 2021. Various plant parts of these plants, Cassia siamea, Dolnix regia, Tamarindus indica and Cassia sieberiana was found effective against egg hatching of M. incognita [25]. These plant show nematocidal effect due to presence of phytochemical such as, phenolics, alkaloids, terpenes, isothiocyanates, tannins thiophenics, glucosides [26]. This has been reported that the leaves of Brassica macrocarpa show nematotoxicity against root knot nematode [27]. After 72 hours of bioassay, the results of an in-vitro mortality experiment revealed that plant extracts of Lindenbergia indica and Scoparia dulcis exhibited the most significant nematicidal action against the survival of the second-stage juveniles. The LC-50 values for Lindenbergia indica extracts at 24, 48, and 72 hours of exposure were lower than for all other treatments. Similar results were obtained by [28] Agbenin et al. 2004, observed that after 24 hours of exposure, all concentrations of dry leaf neem extract caused 100% juvenile mortality. According to [29] Oka et al. aqueous extracts of neem leaves were found to toxicity against M. incognita in vitro. In all treatments, juvenile mortality increases as the concentration increases from 10% to 100% and the exposure period increases from 24 to 72 hours. In all treatments, juvenile mortality increases as the concentration increases from 10% to 100% and the exposure period increases from 24 to 72 hours. As result, it can be concluded that nematode toxicity is dependent on the duration of exposure and the concentration of extracts [30, 31]. Olabiyi [32] also reported that aqueous marigold root extracts were treated to root-knot nematode-infested tomato seedlings, enhanced plant height, leaf and fruit yield and plant leaf and fruit production compared to the control treatment. Elbadri et al. [33] reported that neem leaves extract has chemicals as, aldehydes, phenols, amino acids and fatty acids, terpenes, which are antagonistic to root-knot nematodes. Different strategies were used to inhibit the egg hatching and increased larval mortality of juveniles for nematodes management [34,35]. This has been reported that glycoside (asparagusic acid) obtained from Asparagus officinalis, suppressed Meloidogyne spp. [36]. Recently, two nematicidal chemicals nonacosane-10 ol and 23a-homostigmast-5-en-3b-ol were isolated from the roots of Fumaria parviflora Lam. [37]. Similarly, amended fresh chopped leaves of the five plant species exhibited significant nematicidal potential under greenhouse conditions. This study showed the largest reduction in the number of Egg masses/root, the number of root galls, and final Nematode populations in the soil due to the freshly chopped leaves of Scoparia dulcis treated. Our results are supported by [38, 39] Khan et al. 2019; Khan et al. 2021. According to Chitwood [40] nematicidal characteristics of plant species vary massively based on plant species and cultivar, plant tissue used, plant growth stage, application strategy and nematode species assessed. This significant drop in nematode infestation characteristics might be attributed to compounds found in degraded leaves with ovicidal or larvicidal activities, inhibiting nematode reproduction. The inadequate penetration in the second stage of juvenile and subsequent delays in their feeding and reproductive efforts might ascribe decreased root-knot proliferation. The decline in the number of nematodes might be to account for the plant's increased growth. Limited distractions to the plants result in a well and healthy growth [41]. Oka (2010) [42] observed that the use of plant parts can alter the physical structure and fertility of the soil, resulting in greater plant tolerance to nematode infection in terms of plant growth. The use of botanicals increases plant length (shoot and root) compared to their controls (UIC). There was also significant improvement in the fresh and dry weight of the roots and shoots as a result of the influence of botanical amendment. The above results confirm with khan et al. [43] (2019); [44] khan et al. (2021) and [45] Hasan et al. (2021), in which the nematicidal efficacy of botanicals was tested. This was found increase in tomato growth in treated soils compared to untreated soils might be due to an increase in soil nitrogen availability caused by the addition of botanicals. Adding botanical substances to the soil creates a healthy environment for root development. This increases soil nutrients and releases such toxic compounds, which might significantly minimise the nematode infestation [46,47]. Nitrate reductase (NR) is an important enzyme that functions as a key enzymatic source of nitric oxide in the plant cell. It controls plant development as well as tolerance to abiotic and biotic stress [48]. These findings were similar to reported by Berger et al. [49] found that photosynthesis rates drop when plants come into contact with pathogens. Based on safe, cost-effective and environmentally acceptable methods, organic amendments, plant extracts and bio-pesticides are being utilized primarily [50]. Botanicals in the soil benefited the host plants by fighting the nematode penetration or directly triggering the plant's defensive systems. Resistance or defensive responses are reported in host plants against plant diseases by substances from biocontrol agents and chemicals present in antagonistic plants extracts [51]. The tested aqueous leaf extracts and chopped leaves had significant nematicidal potential against M. incognita. More research is needed to isolate and characterize nematotoxic compounds of these botanicals using advanced techniques to be used in plant-parasitic nematode management in the future instead of hazardous chemical nematicides.

## Conclusion:

Data shows that the botanical extracts examined delay egg hatching and cause mortality of second-stage juveniles of M. incognita. The inhibitory action of extracts is attributed to chemical compounds contained in extracts with ovicidal or larvicidal activities. Data also suggests that applying selected botanicals to the soil as organic amendments function as nematicides and can be successfully utilized to eliminate root-knot nematodes in place of traditional chemical nematicides. Thus, these selected botanicals can be considered to promote organic farming and sustainable management of nematodes. However, further study is needed to investigate the phytochemicals of the selected botanicals that inhibit the nematodes.
